# Contextualizing Engagement With Health Information on Facebook: Using the Social Media Content and Context Elicitation Method

**DOI:** 10.2196/25243

**Published:** 2022-03-04

**Authors:** Yonaira M Rivera, Meghan B Moran, Johannes Thrul, Corinne Joshu, Katherine C Smith

**Affiliations:** 1 Department of Communication School of Communication & Information Rutgers University New Brunswick, NJ United States; 2 Cancer Prevention and Control Program Rutgers Cancer Institute of New Jersey New Brusnwick, NJ United States; 3 Department of Health, Behavior & Society Johns Hopkins Bloomberg School of Public Health Baltimore, MD United States; 4 Department of Mental Health Johns Hopkins Bloomberg School of Public Health Baltimore, MD United States; 5 Department of Epidemiology Johns Hopkins Bloomberg School of Public Health Baltimore, MD United States

**Keywords:** mixed methods, data collection, social media, cancer, health information, Facebook, digital health

## Abstract

**Background:**

Most of what is known regarding health information engagement on social media stems from quantitative methodologies. Public health literature often quantifies engagement by measuring likes, comments, and/or shares of posts within health organizations’ Facebook pages. However, this content may not represent the health information (and misinformation) generally available to and consumed by platform users. Furthermore, some individuals may prefer to engage with information without leaving quantifiable digital traces. Mixed methods approaches may provide a way of surpassing the constraints of assessing engagement with health information by using only currently available social media metrics.

**Objective:**

This study aims to discuss the limitations of current approaches in assessing health information engagement on Facebook and presents the social media content and context elicitation method, a qualitatively driven, mixed methods approach to understanding engagement with health information and how engagement may lead to subsequent actions.

**Methods:**

Data collection, management, and analysis using the social media content and context elicitation method are presented. This method was developed for a broader study exploring how and why US Latinos and Latinas engage with cancer prevention and screening information on Facebook. The study included 20 participants aged between 40 and 75 years without cancer who participated in semistructured, in-depth interviews to discuss their Facebook use and engagement with cancer information on the platform. Participants accessed their Facebook account alongside the researcher, typed *cancer* in the search bar, and discussed cancer-related posts they engaged with during the previous 12 months. Engagement was defined as liking, commenting, and/or sharing a post; clicking on a post link; reading an article in a post; and/or watching a video within a post. Content engagement prompted questions regarding the reasons for engagement and whether engagement triggered further action. Data were managed using MAXQDA (VERBI GmbH) and analyzed using thematic and content analyses.

**Results:**

Data emerging from the social media content and context elicitation method demonstrated that participants mainly engaged with cancer prevention and screening information by viewing and/or reading content (48/66, 73%) without liking, commenting, or sharing it. This method provided rich content regarding how US Latinos and Latinas engage with and act upon cancer prevention and screening information on Facebook. We present 2 emblematic cases from the main study to exemplify the additional information and context elicited from this methodology, which is currently lacking from quantitative approaches.

**Conclusions:**

The social media content and context elicitation method allows a better representation and deeper contextualization of how people engage with and act upon health information and misinformation encountered on social media. This method may be applied to future studies regarding how to best communicate health information on social media, including how these affect assessments of message credibility and accuracy, which can influence health outcomes.

## Introduction

### Background

The rise of health misinformation in today’s social media landscape has prompted a need to better understand how and why individuals engage with this content, as well as its ramifications on health outcomes. Although this topic has gained notoriety in light of the COVID-19 pandemic and its accompanying infodemic, calls for research addressing health misinformation and its unique impact on underserved populations have been present since late 2018 [1]. These calls acknowledge that in addition to defining the prevalence and trends of health misinformation, researchers need to develop approaches that better understand the context of misinformation exchange on social media, the intra- and interpersonal dynamics that influence engagement with content, and how health consequences may stem from these interactions [1].

Reaching populations with evidence-based content through social media has become an important effort to counteract the spread of health misinformation [2,3]. If leveraged correctly, these platforms can be used to encourage participatory communication by fostering user engagement via posts, pictures, videos, and other forms of information sharing [4]. This conceptualization of social media as participatory frames engagement as a way for health organizations to communicate with audiences directly [5] and is typically assessed by evaluating how users respond to content posted on the platform. By playing an active role in conversations about health topics, organizations can also ensure that trust and credibility are established through the dissemination of accurate information [5].

Quantitative methods have undoubtedly helped identify health misinformation trends on social media [6-9]. However, these data are increasingly difficult to obtain [10], do not provide important contextual information regarding what motivates engagement and dissemination among vulnerable populations with poor health outcomes, and cannot capture the effects of misinformation on behavior. Mixed methods approaches that explore the role of these components in the spread of misinformation are necessary to design interventions that minimize and halt dissemination. Mixed methods research comprehensively and purposefully uses both qualitative and quantitative techniques to address an overarching research question that cannot be fully explored and contextualized by either method independently [11]. As such, this paper presents the social media content and context elicitation method, which is a novel approach that incorporates qualitative methods to better contextualize engagement with health information on social media and how this may lead to subsequent actions. This paper first discusses the limitations of the current operationalizations of engagement with health information on social media. This is followed by a detailed description of the social media content and context elicitation method, which was developed to obtain survey data, interviews, and computer screen recordings of cancer-related posts on Latino and Latina participants’ Facebook accounts for quantitative and qualitative analysis. Then, 2 case studies are presented to exemplify the additional information elicited from this methodology, which is currently lacking from other approaches. Finally, we discuss how incorporating qualitative methods, such as those outlined in this paper, allows a better representation of how people engage with health information in reality and provides insights for researchers interested in this type of work.

### Assessing Engagement With Health Information on Facebook

Facebook is among the most popular social media platforms worldwide, with >2.3 billion active users [12]. Second in popularity only to YouTube, 74% of US Facebook users visit the platform on a daily basis [13]. Entertainment, social interaction, and passing time are among the reasons individuals report using Facebook [14]. Facebook has also been a source of health information and social support [15], making it a useful place to engage with general audiences about health topics. Many public health organizations have established a presence on Facebook by creating a Facebook page, which provides a space for businesses and organizations to publicly share information with platform users. Facebook pages provide a direct way for these organizations to deliver evidence-based health information to Facebook users, which is of paramount importance in a social media environment with increasingly unreliable information [3]. Facebook page administrators also have the ability to monitor social media metrics, providing a way for health organizations to operationalize audience engagement with posted content.

Assessing engagement with health-related information on social media is of particular importance as it is a precursor to multiple outcomes, such as increased awareness, knowledge, and behavior change [16,17]. Most studies have assessed engagement by collecting and analyzing data on the likes, comments, and/or shares of posts within an organization’s Facebook page [18-24]. For example, Strekalova and Krieger [24] reported that cancer-related posts on the National Cancer Institute’s Facebook page had a significantly higher number of likes, comments, and shares when they contained images (vs videos, embedded links, or text). Similarly, Srivastava et al [18] found that posts on the American Cancer Society’s Facebook page were more likely to be liked or shared when they contained images or videos, whereas text-based posts were more likely to elicit user comments. Meanwhile, Klippert and Schaper [25] expanded their definition of engagement by including metrics for post reach and clicks on embedded links—both of which are also available to Facebook page administrators. Finally, other studies have captured engagement with cancer information publicly available on Facebook [19,20,26] or Facebook groups [27-30]. Facebook groups differ from Facebook pages in that they can be public or private but do not offer detailed social media metrics and audience insights (although group administrators may extract raw data for analysis through Facebook’s application programming interface). In such cases, engagement has been assessed by quantifying likes, comments, and shares, as these metrics are visible to anyone with access to the posted content.

### Limitations of Quantitative Assessments of Engagement

Measuring engagement with health-related content through these metrics is useful for organizations wanting to assess the success of a social media campaign. It can also provide insight into message factors that may enhance engagement with health information on social media [31]. However, the existing metrics have important limitations. On Facebook, one of these limitations relates to how users are exposed to content. In order for a post from a Facebook page to appear on a person’s news feed, a person must either follow the page or have a Facebook friend who engages with a post from the page. Additional ways users can be exposed to health-related content from a Facebook page are through paid advertising or a Facebook video recommendation, which is based on a video’s popularity or other people and pages a person follows [32]. Even then, the appearance of this content on a person’s news feed is influenced by Facebook’s constantly changing algorithm, which favors content that individuals engage with most often [33]. This has an impact on whether specific health information emerges on a person’s news feed when they log into their Facebook account. As such, engagement with content on a health-related Facebook page may not be emblematic of how the general population engages with such information on Facebook. It is likely that many individuals following a health-related Facebook page are already interested in that particular topic. However, there are many people who may not have an active interest in health information that health organizations are trying to reach, such as healthy individuals who are the target audience for prevention and screening messages. Furthermore, focusing on measuring engagement with evidence-based content posted by health organizations does not fully capture the health information landscape on Facebook, which includes user-generated or shared health misinformation that may not come from reliable sources (eg, a COVID-19–related post dispelling misinformation about vaccine efficiency shared by a Facebook friend with no links to original sources).

Another limitation to quantifying likes, comments, and shares is that these are crude measures of engagement. Although these metrics allow researchers to quantify how some Facebook users visibly engage with health information that is publicly available or posted within a Facebook group, they exclude individuals who do not perform these actions yet still consume health information on the platform [18,24]. Information consumption and lurking—generally defined as reading posts on the web without responding—have been seen as an active and participative form of web-based behavior [34]. Lurking may occur because of environmental, relationship, security, and individual reasons [35]. For example, the quality of a message may be poor (environmental), the user may not feel part of the web-based community (relationship) or have privacy concerns (security), or the person’s needs may be satisfied by just reading a post (individual) [35].

Moreover, although newer Facebook applications, such as CrowdTangle, allow researchers to capture additional engagement metrics (such as post views) [36], these metrics are limited in only establishing general trends with content that is publicly available on the platform. Furthermore, these crude measures fail to capture if and how engagement with health information and misinformation may influence individuals to act upon this information elsewhere. Potential actions may be as small as discussing the information with a friend through messaging apps or as large as incorporating preventive behavior into one’s lifestyle. Understanding these complexities inevitably requires new approaches to help contextualize the impact of engagement on health outcomes.

In response to these needs, we developed the social media content and context elicitation method. This method elicits data concurrently during one-on-one in-person encounters where the participants access their social media profile, scroll through relevant content, and contextualize content engagement with the researcher. In the following sections, we outline the process of collecting, managing, and analyzing elicited data and provide examples of the robust findings that this method provides. We hope that such detail—particularly surrounding data collection and management—enables other scholars to replicate and/or adapt these methods for related studies.

## Methods

### Overview

The methods discussed in this paper were developed for an exploratory, convergent parallel study assessing how and why Latino and Latina adults aged 40 to 75 years without a history of cancer engage with and act upon cancer prevention and screening information or misinformation on Facebook (published elsewhere) [37]. For this study, 20 self-identified Latinos and Latinas aged 40 to 75 years with no history of cancer participated in semistructured, in-depth interviews to discuss their Facebook use and engagement with cancer information on the platform. This diverse population not only avidly uses Facebook but also faces high cancer health disparities: cancer is the leading cause of death among US Latinos and Latinas [38], and cancer incidence rates are highest for screenable cancers linked to preventable behaviors (breast, prostate, and colorectal) [39]. Please refer to the original publication for a full description of the study and the main findings [37].

**Figure 1:**
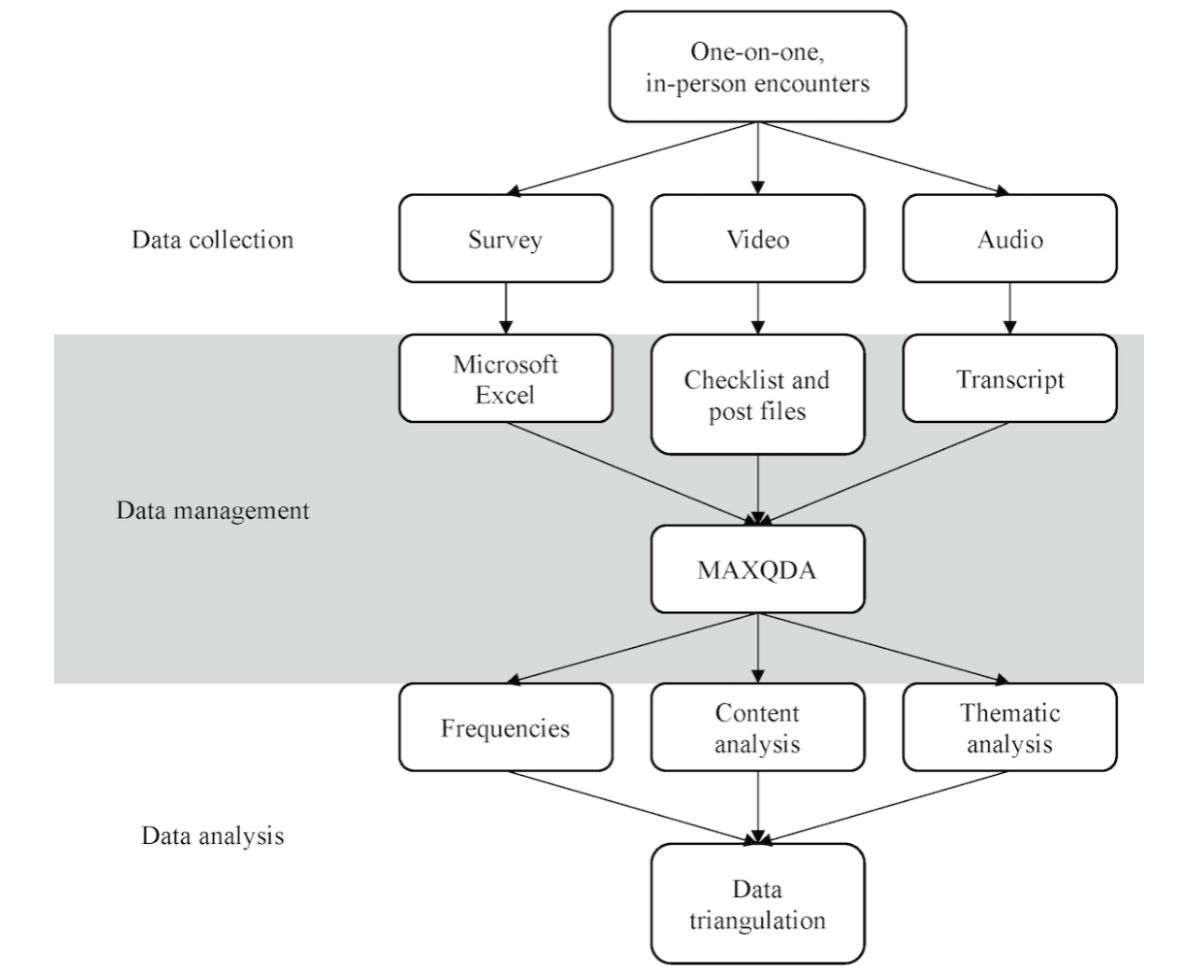
Study design using the social media content and context elicitation method to capture engagement with cancer information on Facebook. Each participant underwent all points of data collection.

The social media content and context elicitation method developed for this study comprised three parts: (1) a short survey collecting demographics, health-related information seeking, and Facebook use data; (2) computer screen recordings of cancer posts appearing on participants’ Facebook during the past 12 months; and (3) semistructured, in-depth interviews discussing Facebook use and engagement with cancer posts on Facebook (Figure 1). Participants were recruited through flyers, word of mouth, and Facebook advertisements. Interviews were conducted in the participants’ language of preference (English or Spanish) by the lead researcher, who is bilingual. All interviews were conducted during the summer of 2018 and lasted approximately 2 hours.

### Data Collection

After providing oral consent, participants completed a short survey collecting demographic variables, basic health-related information seeking, and Facebook use information. This survey provided descriptive insight into the uses and gratifications experienced by Latinos and Latinas on Facebook and other contextual factors that may affect engagement with cancer prevention and screening information on the platform. Following the survey, the researcher began the semistructured interviews, which were audio recorded in their entirety. Using the survey responses as a guide, the researcher asked participants to elaborate on their regular Facebook use patterns and interactions, the extent to which they encountered health information (including cancer information) on Facebook, and what they believed Facebook’s role was in sharing information. Afterward, participants logged into their Facebook account using a private browser on a research laptop and proceeded to turn off the Facebook Messenger feature to avoid being interrupted during the study. The researcher then documented the total number of friends, groups, and pages the participants followed, including how many of these were cancer-related groups or pages.

The participants then went to the search feature on Facebook, which allows Facebook users to search for content posted on the platform. This feature allows users to sort search results using multiple filters, such as *Sort by*, *Posted by*, and *Date posted*. For this study, participants were asked to enter the term *cancer* into the search bar. Once the search results emerged, they were filtered chronologically (*Sort by Most recent*) and by friends and groups the participant followed on Facebook (*Posted by Your friends and groups*). The resulting posts represented all posts that included the word *cancer* that could have potentially appeared on participants’ news feeds when they previously logged into Facebook and corresponded to content either posted by their friends or groups or any other publicly available posts that a friend liked or commented on. The researcher then proceeded to explain the process of jointly scrolling through the past 6 to 12 months of cancer-related posts to discuss posts they recalled seeing and engaging with. Any questions that participants had about the process were discussed before beginning.

Once the participant agreed, the researcher began recording the computer screen using QuickTime Player (version 10.4; Apple), which captures both audio and the computer screen. The researcher and participants jointly scrolled through the content to identify any posts the participants recalled having seen and whether they engaged with the post. Engagement was defined as any combination of the following: liking a post; commenting on a post; sharing a post; clicking on a post link; reading an article in a post; or watching a video within a post. If the post included any video or embedded link, participants were asked if they recalled watching the video or clicking on the link. If so, these were opened to capture the full content.

In addition to capturing the cancer posts that appeared on participants’ Facebook through computer screen recordings, engagement with content prompted the researcher to use a semistructured, in-depth interview guide to ask questions regarding the reasons participants interacted with the post and whether engagement triggered further action. Examples of action included (but were not limited to) searching for additional cancer information or scheduling a cancer screening appointment. In-depth interviews were selected for this study as they allow for the exploration of new issues in depth and elaborate on individuals’ thoughts and behaviors [40], an important facet in exploring how source and content characteristics influence engagement with cancer information on Facebook and any potential subsequent action. Interview guide questions were informed by the Uses and Gratification Theory [41] and the Comprehensive Model of Information Seeking [42,43]. The interview guide covered the following domains: reasons for engagement with cancer information, relationship to the cancer information source, roles of the cancer information source in delivering information on Facebook, perceptions about posted cancer information content and attributes, the ways that source credibility and content accuracy are assessed, and actions triggered by engagement with this information. In cases where participants recalled engaging with a post in ways other than liking, commenting, and/or sharing the post, the participant was asked to elaborate on this type of engagement. The researcher also collected notes regarding each post the participant either recalled or engaged with using a checklist.

Throughout the scrolling process, multiple participants had copious amounts of cancer-related information emerging in their searches, most of which were not specific to prevention and screening topics (eg, survivorship, cancer research, and fundraising). As the purpose of this study was to understand how participants specifically engaged with cancer prevention and screening information, searches were refined midway through the interview. The search terms *cancer prevention* and *cancer screening* were entered in all interviews approximately 30 minutes into the scrolling process to narrow the search results. For each refined search term, the content was scrolled through up to 12 months prior and discussed as previously stated. On several occasions, when guided by the participant and the discussion at hand, additional search terms were added to find specific cancer prevention and screening information participants recalled engaging with. For example, one participant specifically recalled engaging with a post containing information about cancer and soursop (*guanábana*), a Latin American fruit commonly assumed to have curative properties. The post was elicited by searching for *cancer guanábana*. Similarly, another participant recalled a post about cancer diets and asked to search for *cancer diet*. A final search was performed using the term *cancer* and the filter *Posted by you*. This revealed any cancer information posted by the participant on their own Facebook profile.

After discussing the posts, participants were asked wrap-up questions regarding what would make cancer information more appealing on Facebook, who they considered the most influential and trustworthy sources of cancer information among their Facebook friends, and whether Facebook was a source of cancer information they trusted. Notes were taken throughout the interviews and used to inform data management and analysis.

### Data Management

The data collection processes described above elicited rich data: in addition to survey responses, >20 hours of computer screen video and >30 hours of interview audio were captured (Figure 1). Survey responses were entered into a Microsoft Excel spreadsheet. Interview audio recordings were deidentified and transcribed verbatim. The process of capturing discussed posts and deidentifying data recorded on the computer screen is described in the following sections.

The first step in managing all computer screen recordings was to develop a checklist to document all the decision points for each interview video. This checklist collected the time stamps for both the audio and video versions of each interview, which allowed the research team to map interview transcripts with the discussed posts during analysis. Audio and video time stamps were collected at the beginning of the video recording and at the beginning of each post discussed. In addition to marking the time stamps for each post, the checklist was used to summarize the content of each post and to highlight relevant points discussed during the interview. These notes were incorporated as memos associated with each post during the analysis. The checklist was also used to document any search term refinements and outline preliminary codes for subsequent codebook development.

After using the checklist to document each post discussed in the interview, the post was captured through a screen grab and deidentified by cropping and/or covering any identifying images or names with white boxes and saved as a new file identified with the participant’s unique ID; 2 additional files were saved in addition to the post screen grabs when applicable. First, if the post also included a video, the video was captured in its entirety in one of two ways: (1) if the video was part of a publicly available post, the lead researcher recorded the full video by searching for the post on Facebook or (2) if the video was no longer available on Facebook, the segment of the recorded computer screen was trimmed and cropped using iMovie to ensure that the video was deidentified. Second, if a post included a link to an external website that was visited during the interview, the website was captured in one of two ways: (1) if the website link was still accessible, the lead researcher saved a web archive and PDF version of the website page or (2) if the website link was broken or no longer accessible, the recorded segment was deidentified, as described above. All deidentified files (posts, videos, web archives, surveys, and interview transcripts) were saved in a secure cloud-based file sharing and file storage service through the Johns Hopkins University and in an encrypted folder on a password-protected computer. The deidentified data were managed using the MAXQDA (Version 12; VERBI GmbH).

### Data Analysis

The last step was to analyze multiple data elicited through the aforementioned methods. This was performed using traditional data analysis approaches (ie, frequencies, content analysis, and thematic analysis) that were triangulated to explain how and why engagement with cancer prevention and screening content occurred and how this engagement led to further actions. In the following sections, we summarize these analytical approaches; a detailed description of these analyses can be found in the original study [37].

First, we conducted descriptive statistics on all survey data. These findings were used to assist in contextualizing our sample. Then, a content analysis was conducted on all cancer prevention and screening information participants engaged with on their Facebook accounts. Content analysis was used to assess message patterns in a variety of formats, including those available on internet platforms [44]. A codebook was developed using the preliminary codes documented in the checklist during the data management process described in the previous section. The initial coding framework was applied to a sample of 10 cancer posts publicly available on Facebook by the lead researcher and a second bilingual study team member. Discrepancies were discussed and resolved, and a final codebook was developed [37]. Codes were developed for the following areas: post features, post source, post content, and credibility assessment. A total of 2 coders independently coded 10% of the sample. Intercoder reliability was calculated (0.89-1.0) [45], and any discrepancies were discussed until consensus was reached. The lead researcher coded the remaining posts, and code frequencies were calculated upon completion.

Finally, a thematic analysis was conducted on all the interview transcripts. This method allowed for the identification, analysis, and interpretation of patterns or themes in rich interview data sets [46,47], allowing a detailed description of how multiple themes and factors work together to explain engagement with cancer information. Transcriptions were analyzed in their original language to ensure that no meanings were lost in translation. The transcripts were preliminarily coded using emerging codes that aligned with the research questions using a constant comparison method [48]. A coding tree was created to outline the discovered themes and concepts. In addition, memos were composed with exemplary quotes for each theme; any exemplary quotes collected in Spanish were translated into English. Memos were discussed with the study team to ensure dependability and credibility in theme development [49]. The data were placed into larger themes and factors to comprehensively explain how the phenomena occurred. Further data validation was conferred by triangulating the thematic analysis results with those of the content analysis [50] and is discussed in the original paper [37].

### Ethics Approval

This study was approved by the Johns Hopkins School of Public Health institutional review board (IRB8484).

## Results

### Overview

Our study sample comprised 20 self-identified Latino and Latina Facebook users aged 40 to 75 years without a history of cancer (average age 54.2, SD 7.4 years) and represented 7 distinct Latin American subethnic groups from the Caribbean, Central America, and South America; 9 (45%) participants were fully bilingual, 6 (30%) preferred Spanish, and 5 (25%) preferred English. Participants were mainly female (15/20, 75%) and heavy Facebook users, with most (17/20, 85%) reporting checking their Facebook at least once a day. Facebook was most commonly used for social interaction (17/20, 85%) and information sharing (15/20, 75%). Participants had a median value of 357 (IQR 189.5-544.5) Facebook friends and followed a median of 20 (IQR 4.5-56) Facebook groups; only one of the participants followed cancer-related Facebook groups. A detailed description of the sample is available in the main study [37].

Overall, participants reported engaging with 66 posts containing cancer prevention and screening information (4.1 average posts per participant) in the previous year. Data emerging from the social media content and context elicitation method demonstrated that participants mostly engaged with cancer prevention and screening information by viewing and/or reading content (48/66, 73% posts) rather than by liking, commenting, or sharing posts (18/66, 27% posts). Furthermore, it provided rich content regarding how Latinos and Latinas engage with and act upon cancer prevention and screening information on Facebook [37]. In the following sections, we explore 2 sample cases to illustrate how a mixed methods approach provides rich insight that is otherwise missed when quantitative methods are used alone. These 2 cases were selected as they were emblematic of the broad range of information elicited from our sample that goes beyond only quantifying engagement. Participants’ names have been changed to protect their identities.

### Case 1: Rogelio

Rogelio was a bilingual Cuban male aged 61years. He had >1800 Facebook friends and followed 131 Facebook groups, none of which were related to cancer. He considered himself a very active Facebook user, logging in multiple times a day and using the platform for social interactions, searching for and sharing information, seeing what others are doing, and maintaining his cultural identity. During the interview, 13 cancer-related posts were discussed, all of which had a video or image, for he believed that “if it doesn’t enter through the eyes, it doesn’t reach you.” Although he engaged with all 13 posts by reading the content, he did not like, comment, or share any of these on his profile. All but 1 of these posts were shared by friends in his network; the other was shared by a Facebook group to which he belonged. A total of 6 posts were related to natural remedies or foods with curative properties against cancer, 1 was about a free skin cancer screening event, and 1 was about free colorectal and prostate cancer educational sessions for Latino men; the remaining posts were related to cancer survivorship and prayer requests.

**Figure 2:**
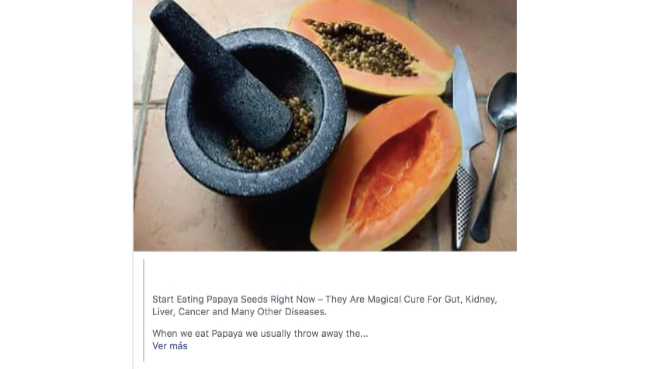
Image of the papaya seed post that Rogelio discussed.

Although Rogelio used his Facebook account frequently throughout the day, he explained that he rarely liked, commented, or shared content on his profile as he could not let others know he was on Facebook during work hours. Therefore, instead of engaging with a post through these metrics, he would send himself interesting posts through Facebook Messenger (the platform’s messaging tool). In this manner, he could read the post at a later time. He also explained how he and his wife regularly shared information related to diet and foods with preventive and/or curative properties through Facebook Messenger. Many times, after discussing content that either one engaged with on Facebook, he would decide whether they would incorporate these natural remedies into their daily lifestyle; he mentioned doing this with the 6 posts discussed during the interview. For example, he described how he and his wife started to eat papaya seeds after he read a post stating that “they are [sic] magical cure for gut, kidney, liver, cancer and many other diseases” (Figure 2). This post described how to consume papaya seeds and outlined 8 benefits, including that papaya seeds “have agents that can stop the growth of tumors and cancer cells, [and] contain isothiocyanate, which helps with breast, colon, leukemia, lung and prostate cancer.”

Rogelio also stated that, although Facebook was one of his main sources of information, he rarely—if ever—verified the information he engaged with on the platform. Instead, he relied on the seriousness of the people who post content on their profiles, stating that his friends from church or those aged >40 years are serious and do not share *fake news*. He also relied on his previous knowledge about a topic and believed that posts about the curative properties of foods are more credible than other topics. For Rogelio, engaging with information through a post was sufficient for him and his wife to incorporate natural remedies into their diets, regardless of whether the post cited an information source.

Finally, his cultural values and Cuban heritage came up frequently during the interview. He tended to have a fatalistic view about cancer, which emerged in multiple discussions. For example, he recalled seeing a post pertaining to 2 educational events for men about colorectal and prostate cancer. When he saw it, he immediately said he never attended such events as speaking about these topics is like inviting the disease into your life:

It’s like not wanting to speak about the topic, so it doesn’t happen to me. As if talking about [colorectal or prostate cancer] puts it in my cabinet.

He believed this avoidance is a very negative Latin American custom; however, he claimed Latinos and Latinas rather *look the other way* when these topics emerge.

### Case 2: Luisa

Luisa was a Puerto Rican female aged 63 years who preferred English. She had 370 Facebook friends and followed 268 Facebook groups, none of which were related to cancer. She also considered herself an avid Facebook user, logging on multiple times a day and using the platform for social interactions, searching for and sharing information, passing time, entertainment, relaxing, expressing her opinions, seeing what others are doing, advocacy, and convenience. During the interview, 11 cancer-related posts were discussed, 5 of which contained cancer prevention and screening information she engaged with. Another 2 posts containing cancer prevention and screening information were discussed as they grabbed her attention during the interview; she had not recalled seeing them previously but stated that she would have read them if she had as they were posted by a friend who she deemed a trustworthy source of health information. The remaining discussed cancer-related posts pertained to cancer survivorship and requests for prayer for survivors of cancer. She shared only 1 post on her profile; she did not like, comment, or share any of the other posts discussed.

When discussing her Facebook use patterns, Luisa stated that she sometimes did not engage through likes, comments, or shares as she was just scrolling through her timeline and did not stop to perform these actions. However, she said this does not mean that she failed to read or watch the content. She gave an example of being at the grocery store line while scrolling through her Facebook: she might watch an interesting video but does not stop to share it with others, only sharing content when “relaxed.”

Luisa was very interested in topics pertaining to cancer prevention, particularly those related to a healthy diet. She discussed superfoods frequently and stated her preference for natural remedies over medication. For example, when discussing a video that included “10 alkaline foods that prevent and treat diabetes, gout, heart disease, and cancer,” she stated that it was the images of different superfoods that initially grabbed her attention, not the cancer prevention claims. She also mentioned that repetition surrounding the benefits of superfoods confirms the credibility of such information. She gave an example of this while discussing engagement with a post about soursop, which stated that it “has been used by many people to fight against cancer cells.” Luisa said that she was familiar with the curative properties of soursop as she had heard this often from friends and family in Puerto Rico. In fact, she had tried to incorporate it into her diet but had not been able to find it in any local supermarket.

Throughout the interview, Luisa continuously mentioned having seen a post about juicing as a way of preventing cancer. She recalled having seen the post on Facebook and copying the recipe on her phone’s notepad app. In discussing this, she also mentioned using Facebook Messenger to send herself articles. At the end of the interview, we were able to find the post by entering the search term *cancer juice*. The post claimed that the super juice recipe “is designed to help us combat breast cancer, as well as helping to starve off all potential cancer cells within the body.” It also stated that the juice cannot be blended as it is a *therapy tonic* that must be prepared using a juicer. The recipe called for broccoli, kale, cauliflower, fresh ginger root, apples, and carrots. She shared that she had since incorporated this juice into her diet, asking for it to be prepared for her when she goes to the supermarket. When asked, she said she decided to include this juice as part of her diet as she considered the friend who posted the recipe to be an extremely trustworthy source of health information. This friend came up 4 times during the interview as she often shared information about natural remedies against many diseases on Facebook, a topic Luisa was very interested in. As Luisa considered this person a trustworthy source of information, she said she rarely further verified the content she posts and might instead just send her any questions through Facebook Messenger. She trusted that her friend had already verified the content shared, although all the websites shared by her friend lacked sources of evidence-based information. When she does decide to verify any information she finds on Facebook, she goes to Google and WebMD.

## Discussion

### Principal Findings

This study presented a qualitatively driven, mixed methods approach to explore how individuals engage with health information on Facebook (specifically, cancer prevention and screening information) and the impact engagement may have on subsequent behavior. In doing so, it expands upon what is known regarding cancer information engagement on social media, which predominantly stems from quantitative methodologies. The current literature operationalizes engagement with information on Facebook through likes, comments, and shares, with some studies further categorizing engagement into levels by type of engagement [16,18-20,25,31]. However, the social media content and context elicitation method adds yet another layer of nuance to public health’s current conceptualization of engagement by providing insight into the different ways people may process and act upon information, particularly individuals who would rather not like, comment, or share posts they consume. As exemplified in the aforementioned case studies, individuals may choose to read, discuss, or even change their behavior based on cancer prevention and screening information they consume without liking, commenting, and/or sharing the information. The aforementioned case studies also show that some individuals may circumvent liking, commenting, and/or sharing by using other messaging platforms to store or share information with others, such as Facebook Messenger and WhatsApp. These findings highlight the importance of exploring how platform interconnectivity affects health information engagement. As such, the presented methodology can assist in developing more comprehensive models describing engagement with health information on social media, responding to calls for a more thorough understanding of engagement on the social media landscape [15].

Consistent with previous literature [35], there are many reasons individuals do not engage with content in ways visible to others on social media. However, this decision is not indicative of a lack of engagement: both cases discussed in this study demonstrate ways in which individuals engage with and even disseminate posts while circumventing likes, comments, and shares. Discounting these aspects of engagement provides a limited explanation of the impact of health information in the social media landscape. This is of paramount importance in the current web-based environment, which is increasingly bombarded with misinformation on a broad range of topics. The social media content and context elicitation method is able to obtain a robust account of how individuals engage with health misinformation, what grabs their attention, how they perceive it, and how they incorporate this information into their daily lives. These insights are necessary to counteract the impact misinformation may have on the uptake of cancer prevention and screening recommendations, which is a growing area of research interest [1]. Although we explore the ramifications of engagement with cancer prevention and screening misinformation in a forthcoming publication, other researchers have already adapted the social media content and context elicitation method to explore the factors related to engagement and disengagement with COVID-19 information on the web [51]. As such, the social media content and context elicitation method may be of particular interest to public health efforts developing social media campaigns targeting misinformation among populations with lower digital and/or health literacy. This method can also provide further insight into features that affect engagement and contribute to the dissemination of accurate cancer information, particularly those conveying prevention and screening recommendations. This method may also be applied to future studies regarding how to best communicate health information on these platforms, an important step toward addressing health disparities.

The process of developing this mixed methodology led to several insights. First, it is important to have a thorough understanding of the social media platform to be explored and its features to maximize how data can be accessed and used for research. In this study, understanding the features that Facebook provides when searching for content on the platform allowed the development of a detailed process to access content alongside participants that may otherwise not be accessible. It also allowed researchers to chronologically discuss content in person with participants, which overrides any algorithms that may affect the visibility of content, while also providing a glimpse to the overall cancer information landscape participants encounter on Facebook. This content not only included cancer prevention and screening information but also information about cancer survivorship, treatment, research, and other cancer topics. In fact, posts with cancer information unrelated to prevention and screening were more common than posts about cancer prevention and screening. Another important observation is that research teams must adapt to the quickly changing nature of social media platforms when embarking on such research efforts. For example, midway during data collection, it was observed that Facebook added a new filter option to their search, which enables users to look only at *Posts you’ve seen*. Although details on how Facebook determines which posts a person has seen are not readily available, including this filter in future research using the methods described in this paper would reduce potential participant recall bias [52].

There are also important ethical considerations researchers must take into account when developing new methodologies to explore content in an increasingly unreliable information landscape on social media. One of these considerations entails privacy concerns. This study took place several months after Facebook’s Cambridge Analytica scandal, where the information of 50 million American Facebook users was used to identify voters’ personalities and influence voting behaviors in the 2016 election [53]. In an additional measure of clarity, the study team developed an additional information sheet for participants that outlined privacy expectations, what data would be captured, and what would and would not be done with captured data once deidentified. It also included images that provided an example of how the discussed posts would be deidentified before analysis. This sheet was discussed in person during the informed consent process and served as a useful resource to ensure participants fully understood the study methods and measures taken to protect the privacy of secondary data. Thus, it is important to be up to date on current events pertaining to social media platforms and issues concerning privacy and other policies that may increase perceptions of mistrust among the general public. It is also important to ensure that potential participants are extremely clear in their understanding of data safeguards in studies that use the aforementioned methods or any other mixed methodologies that capture information from a participant’s social media account or accounts.

This study has several limitations. First, on a practical level, the method described is labor intensive and requires a detailed data collection and management protocol, increasing the resources needed to conduct similar research on a larger scale. This approach may also not be appropriate for more sensitive health topics or individuals who may find these in-depth methods too strenuous. Second, although participants accessed their Facebook accounts on a study laptop, 60% (12/20) of participants reported only accessing their accounts on their cell phones. The visual layout of Facebook’s website version is different from that of its mobile app. This difference in visualization may have affected the ability of some participants to fully recall some posts they previously engaged with as they looked different on the computer screen. Future studies conducting this type of methodology may want to explore using a mobile device to collect data. They may also incorporate the aforementioned new *Posts you’ve seen* filter to minimize recall bias more generally, as self-reported recall may capture only content that people more deeply engaged with rather than all content to which they were exposed and maybe glanced over. Finally, only posts that included the search terms in the text emerged in the search during the data collection process, inevitably excluding posts that did not contain some kind of text feature (eg, posts with only a picture or a direct link to a video). It also excluded posts that discussed cancer-related topics but did not, at minimum, include the word *cancer*, whereas it included posts unrelated to the disease (eg, astrology-related posts or those equating current events in Latin American politics to cancer). Future studies should ensure they possess a comprehensive list of search terms encompassing multiple areas of the study topic while understanding that an increase in search terms adds time to the interview.

### Conclusions

The social media content and context elicitation method shows potential for a deeper contextualization of engagement with health information on social media. Conducting interviews to complement the quantitative content analysis of elicited posts allows a deeper understanding of the reasons and ways engagement with health information on social media occurs, which cannot be done by observing web-based content alone [54] or by asking questions that require recall about a topic that may not be salient to most (ie, cancer prevention and screening information engagement). This mixed methodology also allows a discussion of how message engagement may be a result of offline interactions and relationships and how these affect assessments of message credibility and accuracy. Our findings provide insight into the preferred source and content characteristics of information on social media that triggers engagement and subsequent action among specific groups and vulnerable populations, laying foundational work for the development of future measures and empirical research exploring innovative and participatory health communication on social media platforms. Future steps for the research described in this paper include data integration and the development of a final conceptual model to help visualize the process of engagement with cancer prevention and screening information on Facebook among Latinos and Latinas in the United States.
